# GC-MS Combined with Proteomic Analysis of Volatile Compounds and Formation Mechanisms in Green Teas with Different Aroma Types

**DOI:** 10.3390/foods13121848

**Published:** 2024-06-13

**Authors:** Xiaojun Niu, Cun Ao, Jizhong Yu, Yun Zhao, Haitao Huang

**Affiliations:** Tea Research Institute, Hangzhou Academy of Agricultural Science, Hangzhou 310024, China; xiaojunwords@126.com (X.N.); aocun123@163.com (C.A.); jz310024@126.com (J.Y.); yunz2431@163.com (Y.Z.)

**Keywords:** baked green tea, tender aroma, floral-like aroma, volatile component, proteomic

## Abstract

Aroma is one of the key factors for evaluating the quality of green tea. A tender aroma (NX) and floral-like aroma (HX) are two types of high-quality aroma of green tea. In this work, the different aroma types of baked green tea were classified by sensory evaluation. Then, seven tea samples with a typical tender or floral-like aroma were selected for further volatile component analysis by GC-MS. A total of 43 aroma compounds were identified in two different aroma types of baked green tea samples. The PCA showed that linalool, geraniol, 3-hexenyl butyrate, and 3-hexenyl hexanoate were the major volatiles contributing to the HX. On the other hand, most of the alcohol volatiles, such as 1-octanol, 1-octen-3-ol, 1-dodecanol, 1-hexadecanol, phenylethyl alcohol, benzyl alcohol, aldehydes and some hydrocarbons contributed more to the NX. In addition, the chemical composition analysis showed that the content of free amino acids was higher in NX green tea samples, while the content of catechins was relatively higher in HX tea samples. A proteomic analysis revealed that most of the enzymes involved in VPBs pathways, such as phenylalanine ammonialyase, peroxidase, and shikimate-O-hydroxycinnamoyl transferase, were more abundant in NX than in HX tea samples. These results laid a foundation for the aroma formation mechanism of different aroma types of baked green tea and provided some theoretical guidance for the breeding of specific aroma varieties.

## 1. Introduction

Tea is one of the world’s most popular beverages. Based on the degree of fermentation by endogenous enzymes in the tea leaves, tea can be classified into three main categories, including green tea (non-fermented), oolong tea (semi-fermented), and black tea (fully fermented) [1]. Of these, green tea is also the most widely consumed tea in most East Asian countries (especially China) due to its delightful flavor, many health benefits, and a long cultural history. In 2022, the tea plantation area in China was approximately 3.2641 million ha, with a yield of 3.1810 million tonnes, of which green tea (1.8538 tonnes) accounted for 58.3% of the total output. Meanwhile, its production value accounts for 64.71% of the total tea production in China [2]. According to different manufacturing processes, green tea can generally be divided into four categories: baked green tea, roasted green tea, steamed green tea, and sun-dried green tea [3]. There are significant differences in the flavor and other quality characteristics of different categories of green tea due to the tea plant variety, growing conditions, and processing technology.

Aroma is one of the key factors in judging the quality and character of tea, and it also influences consumer choice. The type of aroma characteristics of green tea can be classified into various aroma types, such as clean and refreshing (Chinese: Qingxiang, QX), chestnut-like (Chinese: Lixiang, LX), tender (Chinese: Nenxiang, NX), and floral-like (Chinese: Huaxiang, HX) aromas, among others, categories based on sensory characteristics [4,5,6]. It is well known that the aroma type of tea is a comprehensive effect of numerous aroma components, which is determined by its chemical composition. The formation of different aroma types is mainly due to the comprehensive effects of different types and concentrations of aroma compounds on the human olfactory nerves.

Recently, over 700 volatile components have been identified from various types of tea, including alcohols, aldehydes, ketones, esters, lactones, acids, phenols, heterocyclic compounds, sulfur compounds, nitrogen compounds, and hydrocarbons, among others [7]. Although over 300 aroma components have been identified in green tea, not all of them are involved in the formation of tea aroma quality and tea aroma type. Only a few key aroma components play an important role, such as alcohols (linalool and its oxides, geraniol, nerolidol, etc.), aldehydes (decanal, hexanal, nonanal, etc.), ketones (1-octen-3-one, β-ionone, etc.), esters (capro-cis-3-hexelate, methyl salicylate, methyl jasmonate, etc.) and so on [3,8,9]. Therefore, the identification of the key volatile compounds in different flavors of green tea is particularly relevant.

During the past decade, gas chromatography–mass spectrometry (GC-MS) has become the most widely used analytical technique for the detection of volatile components in teas and other foods, due to its rapid qualitative and accurate quantitative capabilities [10,11,12]. Moreover, the methodology of headspace solid-phase microextraction combined with gas chromatography–mass spectrometry (HS-SPME/GC-MS) has also been widely applied for the analysis of volatile and semi-volatile compounds in environmental, food, and biological samples, due to its ease of operation and good extraction performance [13]. Numerous aroma compounds have been identified using this technology, to elucidate the formation of different types of tea aroma in green tea [7,8,14,15]. In recent years, omics technology has attracted researchers in the field of food and nutrition, because it can reveal complex biochemical processes and regulatory mechanisms in living organisms. It has been widely used in the field of food flavor analysis [16,17,18]. Moreover, the use of multi-omics combined analysis technology to reveal the flavor of tea has also become an effective method [19]. The aim of this work was to elucidate the major volatile compounds contributing to the specific aromas of baked green tea, including NX and HX. Firstly, the aroma type classification of tea samples made from different varieties was carried out through sensory evaluation. Then, the volatile components, chemical compositions, and the differences of related metabolic pathway enzymes of tea samples with different aroma types were identified using GC-MS, PCA, and label-free proteomic techniques.

## 2. Materials and Methods

### 2.1. Tea Samples

A total of 26 tea samples were used in this study. Fresh tea leaves were picked based on the picking standards of a bud with one leaf in the spring of 2017 (10 samples) and 2019 (16 samples) at the experimental base of Daqing valley, the Tea Institute, Hangzhou Academy of Agricultural Sciences. Then, the manufacturing process of baked green tea samples was performed according to the following process: firstly, the fresh tea leaves were withered for 4–6 h, and then they were fixed by a flat-shape tea-fixing machine to terminate the activities of the endogenous enzymes at 200–230 °C for about 2.5 min. They were allowed to cool and rolled manually for 10 min. Finally, they were dried in a dryer at 100 °C for 12 min and then at 80 °C until the moisture content was about 5%. Some of the fresh tea leaves were freeze-dried in liquid nitrogen for proteomic analysis, and the rest of the fresh tea leaves were processed into the corresponding baked green tea samples using a consistent processing method. The fresh leaves and dried tea samples were stored in a refrigerator at −80 °C and −20 °C, respectively, until further analysis.

### 2.2. Sensory Evaluation

The sensory evaluation was carried out at the Sensory Analysis Unit of the Tea Institute, Hangzhou Academy of Agricultural Sciences, China. The aroma characteristics of the tea samples were described based on the Chinese national standard in “Methodology of sensory evaluation of tea” (GB/T 23776—2018) by three well-trained tea experts. The specific steps of the sensory evaluation are mainly based on the description of Zhu et al. [20]. The results of the sensory evaluation were based on the principle of minority rule.

### 2.3. Chemicals and Standards

Folin phenol, anhydrous sodium carbonate, potassium dihydrogen phosphate, and disodium hydrogen phosphate were purchased from Sinopharm (Shanghai, China). Acetic acid, acetonitrile, ethylenediaminetetraacetic acid disodium salt, and L-ascorbic acid were purchased from Merck KGaA, Darmstadt, Germany. Catechin monomers and theanine standards were purchased from Sigma-Aldrich (Shanghai, China). Distilled water was purchased from the Wahaha Group Company (Hangzhou, China). Solid-phase microextraction (SPME) fiber (CAR-DVB-PDMS), 50/30 μm, with a length of 2 cm, was purchased from Supelco (Bellefonte, PA, USA). Standard samples of catechins, including catechin (C), epicatechin (EC), gallocatechin (GC), epigallocatechin (EGC), catechin gallate (CG), epicatechin gallate (ECG), epigallocatechin gallate (GCG), epigallocatechin gallate (EGCG), and caffeine (CAF), were purchased from Sigma-Aldrich (Shanghai, China).

### 2.4. Chemical Composition Analyses

An estimation of the total free amino acid content in the tea leaves was determined using the ninhydrin method described by Chen et al. [21]. The total polyphenol content was determined by the Folin colorimetric method described by Teng et al. [22]. Catechin components and caffeine were estimated using a high-performance liquid chromatography (HPLC, Agilent 1100, Santa Clara, CA, USA) system equipped with a Hypsial ODS C18 column (4.6 mm × 250 mm, 5 µm). The detection wavelength and column temperature were set at 280 nm and 35 °C, respectively. Mobile phase A (acetic acid–deionized water, 1:49) and B (acetonitrile 100%) were run in linear gradients, with A decreasing from 93.5% to 85% in the first 25 min, A decreasing from 85% to 75% in the next 5 min, and then A increasing from 75% to 93.5% in 3 min and maintained for 2 min. Each injection volume was 10 µL, and the flow rate was 1.0 mL/min. The content of the different components was quantified based on the peak area using the external standard method. The external standard samples included C, EC, GC, EGC, CG, ECG, GCG, EGCG, and CAF. The experimental data were calculated using three independent replicates.

### 2.5. Determination of Volatile Compounds

#### 2.5.1. Extraction of Aroma Compounds

Accurately, 3 g of tea sample was weighed and placed in a 250 mL conical flask, and 150 mL boiling water was added. Subsequently, the conical flask was sealed with tin foil and immersed in a 70 °C water bath for 10 min, after which the tea broth was filtered with 4 layers of gauze and transferred to another conical flask. After cooling to room temperature, 10 mL of tea broth was removed from the conical flask and transferred to a 20 mL headspace flask. Then, 5 µL of the internal standard (ethyl decanoate 25 mg/L) and 2 g of sodium chloride was added into the headspace flask and subsequently shaken. The headspace bottle cap was immediately tightened and placed in a thermostatized water bath to equilibrate for 10 min at 60 °C, and then the SPME fiber was exposed to the sample vial headspace for 40 min.

#### 2.5.2. GC-MS Analysis

A TRACE™ 2000 gas chromatograph equipped with a Trace DSQ mass spectrometer (Thermo Finnigan, San Jose, CA, USA) was used. Desorption was performed on a GC analyzer in splitless mode at 250 °C for 5 min. Volatile separation was performed using an HP-INNOWAX column (30 m × 0.32 mm × 0.50 μm, Agilent, USA). The oven temperature programmer was initially set at 50 °C (held for 5 min), then ramped at 3 °C/min to 210 °C (held for 5 min), then increased at a rate of 15 °C/min to 230 °C (held for 5 min). The carrier gas was helium (>99.999%) at a constant flow rate of 1.0 mL/min. Mass spectrometer conditions: Ion source temperature, 250 °C. Interface temperature, 230 °C. The mass spectrometer was run in a scanning range from 35 to 540 *m*/*z* at 70 eV.

#### 2.5.3. Identification and Quantification of Aroma Compounds

Volatile compounds were tentatively identified by comparing their mass spectra with the NIST mass spectrometry library, retention index (RI), and by comparing the peak type and peak emergence time with data available in the literature [23]. The relative content of volatile compounds was calculated using the formula of A_i_/A total, where A_i_ represents the peak area of each volatile compound and A represents the total peak area of all volatile compounds in a single sample.

### 2.6. Proteomic Analysis

#### 2.6.1. Protein Extraction

Total protein was extracted as described by Wisniewski et al. with slight modifications [24]. About 0.1 g of frozen fresh leaves was ground to powder using liquid nitrogen. The tissue was transferred to 2 mL Eppendorf tubes and kept frozen, then 1.5 mL protein extraction solution I (40 g trichloroacetic acid dissolved in 400 mL protein extraction solution II) was added to the tube, extracted at −20 °C for 1 h, shaken 5~6 times uniformly, and spun at 13,000 rpm at 4 °C for 20 min. The supernatant was removed and 1.5 mL protein extraction solution II (500 mL acetone and 350 µL β-mercaptoethanol) was added, extracted at −20 °C for 1 h, and spun at 13,000 rpm at 4 °C for 20 min. The above steps were repeated twice. The precipitates were washed twice with 90% ice-cold acetone and dried under vacuum to obtain dry protein powder (the drying time is about half an hour). Approximately 20 mg of protein powder was solubilized in protein lysate solution (8 M urea and 4% sodium dodecyl sulphate (SDS)) at room temperature for about 1 h, and shaken 5~6 times evenly during this time. After centrifugation at 13,000 rpm for 30 min, the protein supernatants were filtered through a 0.22 µm membrane. The final protein filtrates were quantified using a bovine serum albumin protein assay kit (Thermo Fisher Scientific, Waltham, MA, USA) and determined by SDS–polyacrylamide gel electrophoresis (SDS-PAGE). Proteins were stored at −80 °C until further analysis.

#### 2.6.2. Protein Digestion and Deionization

A total of 150 μg of protein samples was placed in a 1.5 mL EP tube, and 100 mM NH_4_HCO_3_ was added to make it up to 100 µL. Then, 11 µL of 100 mM dithiothreitol (DTT) was added to a final concentration of 10 mM, gently vortexed, and incubated in a metal bath at 37 °C for 1 h. Then, 12 µL of 500 mM iodoacetamide (IAA) was added to the tube. Samples were incubated at room temperature for 30 min in the dark and transferred to an ultrafiltration filter (10 kD, Sartorius; Göttingen, Germany), centrifuged at 12,000× *g* for 15 min at 4 °C, and the filtrates were discarded. A total of 150 µL of 100 mM NH_4_HCO_3_ was added to the ultrafiltration and centrifuged twice at 12,000× *g* for 15 min at 4 °C. Finally, proteins were digested with 6 μg trypsin (protein–trypsin = 25:1) in 100 µL 100 mM NH_4_HCO_3_ at 37 °C for 12–16 h. After trypsin digestion, the peptide segments were dried using a vacuum pump.

#### 2.6.3. Peptide Desalination and Quantification

Peptides were eluted with 5% trifluoroacetic acid (TFA) solution, until the final TFA concentration was 0.1–1%. The peptides were then desalted on C18 filter cartridges (EmporeTM SPE Cartridges C18, 7 mm internal bed diameter, 3 mL volume, Sigma, St. Louis, MO, USA) and washed three times with 0.1% formic acid (FA). The wash components were collected by vacuum centrifugation and a step gradient elution was started. The lyophilized sample was redissolved in 20 µL of 0.1% FA, and the peptide concentration was measured by OD_280._

#### 2.6.4. Liquid Chromatography–Mass Spectrometry Analysis

QExactive HF-X mass spectrometry, combined with Easy nLC (Thermo Fisher, USA), was used for the LC-MS/MS analysis. The detailed steps of the LC-MS/MS analysis are based on the report by Chen et al. [25].

Mass spectrometry data analysis: Raw file data were analyzed using Maxquant software (version 1.6.0.1) for label-free qualitative and quantitative analysis, and protein databases of species such as Prunus, Rosaceae, and green plants were searched. All differentially expressed proteins (DEPs) were mapped to the Gene Ontology (GO) database (http://www.geneontology.org/, accessed on 4 April 2024) for functional annotation. A Kyoto Encyclopedia of Genes and Genomes (KEGG) pathway enrichment analysis (http://www.genome.jp/kegg/, accessed on 4 April 2024) was performed using the KEGG Automatic Annotation Server.

### 2.7. Statistical Analysis

Statistical analysis was performed by SPSS software (version 19.0) and Microsoft Excel 2019. The measured content of the aroma compounds was expressed as mean ± standard deviation (SD). Student’s *t*-test was used to determine the differences between the different tea samples at a 5% and 1% significance level, respectively. Principal component analysis was performed using Origin 8.0 (MicroCal, Northampton, MA, USA). Proteomic data analysis was performed using the Meiji Biological Cloud platform (https://cloud.majorbio.com/, accessed on 4 April 2024).

## 3. Results and Discussion

### 3.1. Sensory Evaluation Results for the Baked Green Tea Samples

The tea plant variety is one of the key factors determining the aroma of tea. As shown in Table 1, the different tea samples exhibited different aroma characteristics (such as high brisk, tender aroma, clean aroma, and floral-like, etc.) when using the same manufacturing process. Four of the tea samples showed a typical tender aroma, namely, Fuding, Lifeng, 741103, and 741006. Three of the tea samples, 730206, 730120, and 741209, had a typical floral-like aroma. These seven tea samples were selected for further aroma component analysis.

### 3.2. Detected Volatile Compounds in Baked Green Teas by GC-MS

**Figure 1:**
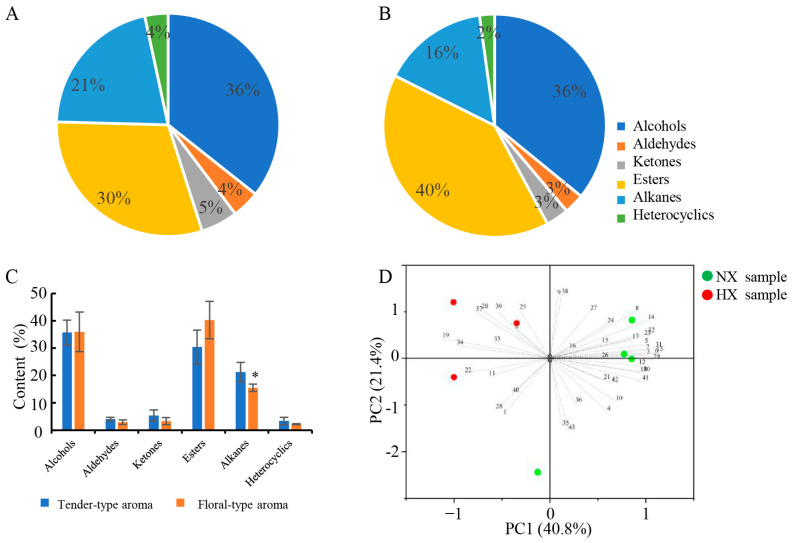
Analysis of aroma components in different aroma-type tea samples. (**A**) Proportion of aroma components in NX tea samples. (**B**) Proportion of aroma components in HX tea samples. (**C**) The relative content of aroma components in two different aroma-types of tea samples. * Indicates significant difference (*p* < 0.05) in *t*-test. (**D**) Principal component analysis of aroma components in two different aroma-types of tea samples. The number represents the code of aroma components.

The volatile compounds in each tea sample were analyzed by HS-SPME/GC-MS, and the major volatile components and content are presented in Figure 1 and Table S1. A total of 43 volatile compounds were detected and tentatively identified, including 9 alcohols, 5 aldehydes, 4 ketones, 14 alkanes, 10 esters, and 1 heterocyclic compound. As shown in Figure 1A,B, the composition of volatile compounds in two aroma types of baked green tea was similar, but the aroma content differed slightly. Alcohols were the richest volatile compounds in NX green tea samples (average of 36%), followed by esters (average of 30%). In HX green tea samples, esters were the most abundant volatile compounds (average of 40%), followed by alcohols (average of 36%). The total content of alcohols and esters was more than 60% in the two aroma types of baked green tea, but the aldehydes, ketones, and heterocyclic compounds were relatively low. In addition, there was a significant difference in the relative content of hydrocarbons between the two different aroma types of baked green tea (Figure 1C).

As shown in Table S1, there were significant differences in the relative content of different volatile compounds in the baked green tea samples. Among them, linalool had the highest content of tea aroma compounds in these tea samples, while there was no significant difference between the two types of tea samples. The content of linalool in HX tea samples was slightly higher than that in NX tea samples. Linalool is a colorless liquid with a strong grassy, sweet woody, floral, and citrus-like aroma. Several previous studies have revealed linalool as a key volatile of specific aroma types in green tea [20,26,27,28]. 3-hexenyl hexanoate had the second highest content of tea aroma components in HX tea samples; the average content ranged from 8.59 to 16.07%, significantly higher than in NX tea samples (average of 5.68 to 9.11%). Xue et al. reported that 3-hexenyl hexanoate contributes to the formation of the floral flavor in black tea [29]. In addition, 1-dodecanol, nonanal, and many esters also promoted the formation of the unique floral aromas in green teas [30,31,32].

In recent years, there have been few studies on the mechanism of aroma formation in NX green tea. Zhou et al. found that the tender aroma characteristics of Longjing tea were significantly positively correlated with the content of compounds such as styrene, hexadecane, and 3-methoxy-1,2-propanediol in the aroma components [33]. Zheng et al. found that linalool oxide, 3-hexenyl butyrate, and 3-hexenyl isovalerate can be used as characteristic volatile components of tender-aroma green tea [34]. In this study, 3-hexenyl butyrate and 3-hexenyl hexanoate showed significant differences in the two different aroma types of green tea. However, the levels of these two aroma compounds were higher in the HX tea samples than in the NX tea samples. Furthermore, there was no significant difference in the content of hexadecane between the two different-flavored tea samples, but its content was higher in the NX tea samples than in the HX tea samples.

In addition, some low-content aroma compounds such as α-cubebene also showed significant differences in the two different aroma types of baked green tea (Table S1). These results indicate that these low levels of aroma compounds may play a key role in distinguishing different aroma types of tea samples. Previous studies have also reported that these low-content volatile compounds also play a crucial role in the formation of green tea aroma [28,35,36,37]. Therefore, the formation of tea aroma is not only related to the content of aroma compounds and aroma components, but also the odor threshold values of each determined compound, as well as their odor activity value (OAV), were also very important factors.

### 3.3. PCA

To explore the differences in aroma compounds in different aroma types of baked green tea, a principal component analysis (PCA) was performed on the content of aroma components. As shown in Figure 1D, there was a clear separation in the different aroma types of the tea samples, indicating that the volatile profile of the NX tea sample was quite different from that of the HX tea sample. The first principal component (PC1) accounted for 40.8%, and the second principal component (PC2) accounted for 21.4%. As shown in Figure 1D and Table S1, 1-octanol, 1-octen-3-ol, 1-dodecanol, 1-hexadecanol, phenylethyl alcohol, benzyl alcohol, nonanal, decanal, β-cyclocitral, β-ionone, hexadecane, 3-hexenyl isovalerate, etc., mainly contributed to PC1. Meanwhile, linalool, geraniol, benzaldehyde, 3-hexenyl butyrate, 3-hexenyl hexanoate, 3-hexen-1-ol acetate, 3-hexenyl-3-hexenoate, hex-3-enyl-2-methylbut-2-enoate, 3-carene, d-cadinene, α-cubebene, and α-muurolene contributed strongly to PC2. PC2 contains mainly HX-type samples. Liu et al. suggested that 1-hexanol, linalool oxide, linalool, geraniol, β-ionone, isoamyl acetate, and 2-methylpropanal were significantly enriched in flowery-type tea and contributed to the floral-like aroma [14]. β-ionone, linalool, geraniol, methyl salicylate, and other components may jointly contribute to the floral aroma of baked green tea [28]. Wang et al. reported that volatile components such as linalool, furfural, β-ionone, hexanoic acid-3-hexene ester, ethylbenzene, naphthalene, and 2-n-pentylfuran made important contributions to the aroma quality of fresh-scented green tea [38]. Our results also showed that linalool, geraniol, 3-hexenyl butyrate, and 3-hexenyl hexanoate were important volatiles contributing to a floral-like aroma. In addition, according to PC1, most of the alcohol aroma components, such as 1-octanol, 1-octen-3-ol, 1-dodecanol, 1-hexadecanol, phenylethyl alcohol, benzyl alcohol, aldehydes, and some hydrocarbons, contributed more to the tender aroma.

### 3.4. Chemical Compositions Detected in Different Flavors of Baked Green Tea

**Figure 2:**
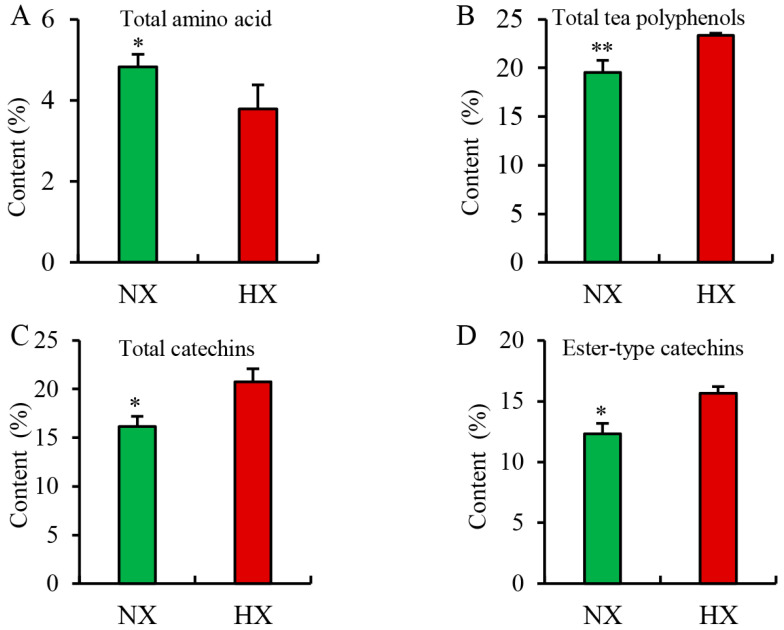
The relative content of representative chemical compositions in two different aroma types of baked green tea samples. * and ** indicate significant difference (*p* < 0.05) and extremely significant difference (*p* < 0.01) in *t*-test, respectively. (**A**) Total amino acid; (**B**) Total tea polyphenols; (**C**) Total catechins; (**D**) Ester-type catechins.

To investigate whether there were differences in the chemical composition of different flavors of baked green tea, the chemical composition content was analyzed. As shown in Figure 2 and Table S2, the content of total amino acids in NX tea samples (average of 4.42 to 5.15%) was significantly (*p* < 0.05) higher than that in HX tea samples (average of 3.15 to 4.30%). Meanwhile, the content of tea polyphenols in NX tea samples (average of 17.72 to 20.46%) was lower in HX tea samples (average of 23.17 to 23.59%). In addition, there were significant differences in the levels of total catechins, simple catechins, and ester-type catechins between the two different aroma types of baked green tea. The levels of catechins, simple catechins, and ester-type catechins in the HX samples were higher than those in the NX samples. Amino acids are thought to play a major role in making green tea taste refreshing. During tea processing, amino acids may also undergo decarboxylation or deamination reactions, or Maillard reactions with sugars, to generate new flavors and aromas [39]. In addition, the content of catechins, especially epigallocatechin gallate and epigallocatechin, play a key role in the taste and aroma quality of green tea [40]. Therefore, our results indicated that tea varieties with a high content of free amino acids are suitable for the production of NX green tea, while tea varieties with a high content of catechins are suitable for the production of HX green tea.

### 3.5. Proteomic Analysis

**Figure 3:**
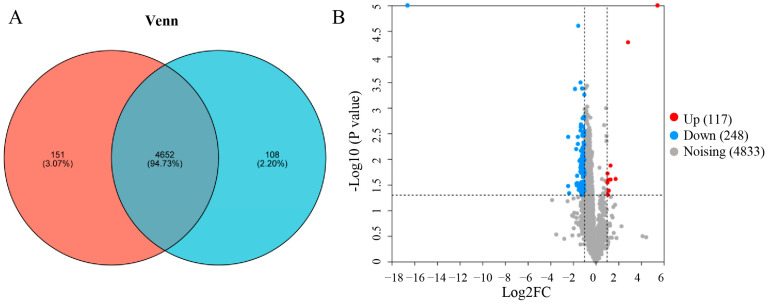
The number of proteins identified in the study. (**A**) The number of proteins identified in different aroma types of tea samples. (**B**) DEPs as a volcano plot in different aroma types of tea samples.

Sixteen tea samples exhibiting a typical tender aroma and floral-like aroma were selected for proteomic analysis in 2019 (Table S3). A total of 4911 related proteins were identified in this study, of which 4652 proteins were detected in two different aroma types of baked green tea samples (Figure 3A). Fold modification values larger than 2.0 times and a *p* value < 0.05 (from Student’s *t*-tests) were used to identify differentially expressed proteins (DEPs). As shown in Figure 3B and Table S4, 365 proteins showed significant differences between NX tea samples and HX tea samples, including 117 up-regulated and 248 down-regulated proteins.

**Figure 4:**
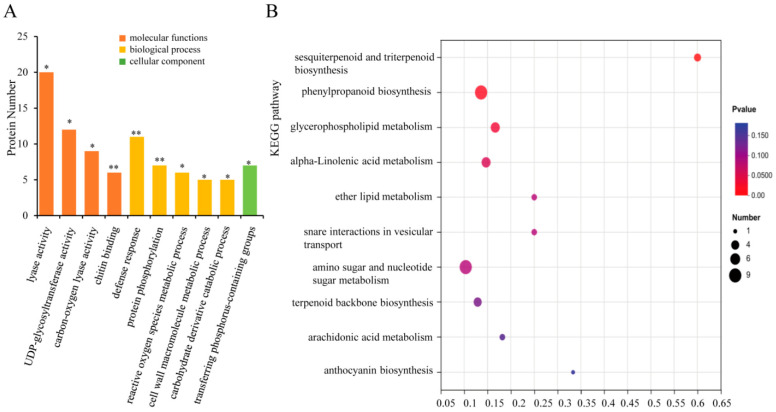
GO (**A**) and KEGG (**B**) analysis of differentially expressed proteins. * and ** indicate significant difference (*p* < 0.05) and extremely significant difference (*p* < 0.01), respectively.

A bar graph of the top 10 GO enrichment results is shown in Figure 4A and Table S5. The results showed that the defense response, protein phosphorylation, reactive oxygen species metabolic process, cell wall macromolecule metabolic process, and carbohydrate-derivative catabolic process were significantly enriched terms of biological processes. Lyase activity, UDP-glycosyl transferase activity, carbon-oxygen lyase activity, and chitin binding were mainly involved under the molecular functions category. In addition, transferase complexes, transferring phosphorus-containing groups, were significantly enriched proteins under the cellular component category. Among these, the most dominant categories were lyase activity, UDP-glycosyltransferase activity, and the defense response. Moreover, as shown in Figure 4B and Table S5, the top 10 enriched KEGG pathways of these DEPs were mainly involved in sesquiterpenoid and triterpenoid biosynthesis; phenylpropanoid biosynthesis; glycerophospholipid metabolism; alpha-linolenic acid metabolism; ether lipid metabolism; snare interactions in vesicular transport; amino sugar and nucleotide sugar metabolism; terpenoid backbone biosynthesis; arachidonic acid metabolism and the anthocyanin biosynthesis signaling pathway. Among these pathways, sesquiterpenoid and triterpenoid biosynthesis, phenylpropanoid biosynthesis, and glycerophospholipid metabolism-related pathways were most enriched. Sesquiterpenes, terpenes, and their derivatives are important volatile secondary metabolites in plants; these compounds often play a decisive role in determining the type of tea aroma [41,42]. A recent study also showed a positive correlation between tea tree varieties and linalool content [43]. As shown in Table S5, three DEPs were directly involved in the metabolic pathways of ko00909 (sesquiterpenoid and triterpenoid biosynthesis), ko00100 (steroid biosynthesis) and ko01110 (biosynthesis of secondary metabolites), including GERD, FDFT1, and LUP4.

Moreover, volatile phenylpropanoids/benzenoids (VPBs) are also important contributors to the formation of tea aroma [44]. Research has found that most VPBs belong to the shikimic acid metabolism pathway. The first step, which plays a crucial role in this metabolic pathway, is the formation of cinnamic acid from L-phenylalanine, catalyzed by phenylalanine lyase [45]. As shown in Table S5, nine DEPs were involved in phenylpropanoid biosynthesis, including ko00360 (phenylalanine metabolism), ko00940 (phenylpropanoid biosynthesis), ko00941 (flavonoid biosynthesis) and ko00945 (stilbenoid, diarylheptanoid, and gingerol biosynthesis). Most of the enzymes involved in these pathways, such as phenylalanine ammonia-lyase, peroxidase, shikimate-O-hydroxycinnamoyl transferase, and coniferyl aldehyde dehydrogenase, were more abundant in NX tea samples than in floral HX tea samples. This suggests that the high expression of enzymes involved in phenylpropanoid biosynthesis may contribute to the release of high-intensity aroma compounds. The major phenylpropanoids in tea are phenylethanol and phenylacetaldehyde [46]. As shown in Table S1, the relative content of phenylethanol was higher in NX tea samples (average of 2.59%) than in HX tea samples (average of 0.54%). Therefore, the expression levels of the above enzymes in fresh tea leaves can be used as indicators in future work for breeding specific aroma varieties.

## 4. Conclusions

In this study, a total of 26 tea varieties/samples were processed into baked green teas using a consistent process, and the samples were subjected to sensory evaluation. From these, seven tea samples with typical aromas were selected for further aroma component analysis. The result of the PCA showed that linalool, geraniol, 3-hexenyl butyrate, and 3-hexenyl hexanoate were the major volatiles contributing to the floral-like aroma. Meanwhile, the major alcohol aroma components such as 1-octanol, 1-octen-3-ol, 1-dodecanol, 1-hexadecanol, phenylethyl alcohol, benzyl alcohol, aldehydes, and partly hydrocarbons contributed more to the tender aroma. Furthermore, it was found that tea varieties with a high content of free amino acids may be suitable for the production of NX baked green tea, while tea varieties with high levels of catechins are suitable for the production of HX baked green tea. The proteomic analysis showed that most of the enzymes involved in VPBs pathways, such as phenylalanine ammonia-lyase, peroxidase, and shikimate O-hydroxycinnamoyl transferase, were higher in NX tea samples than in HX tea samples. Therefore, the expression levels of these enzymes in fresh tea leaves can be used as indicators in future work to breed specific aroma varieties.

## Figures and Tables

**Table 1:** Aroma sensory evaluation results of baked green teas in 2017.

NO.	Cultival	Aroma Characteristics
1	Fuding	High brisk, with tender aroma
2	Lifeng	Clean and refreshing, tender aroma
3	741103	High brisk, obvious tender aroma
4	741006	Obvious tend aroma and tip aroma
5	J1130	Clean and refreshing, fresh and brisk
6	730206	Fragrant and lasting
7	730120	With flowery aroma
8	741209	Obvious flowery aroma
9	XGL16-14	High brisk
10	740209	High brisk, with tip aroma

## Data Availability

The original contributions presented in the study are included in the article, further inquiries can be directed to the corresponding authors.

## Supplementary Materials

The following supporting information can be downloaded at: https://www.mdpi.com/article/10.3390/foods13121848/s1, Table S1: Aroma components and content in different aroma-type baked green tea samples; Table S2: Analysis of chemical compositions in different aroma-type baked green tea samples; Table S3: Aroma sensory evaluation results of different baked green teas in 2019; Table S4: Differential proteins in two groups of baked green tea with different aroma types. Table S5: GO and KEGG pathway analysis of differentially expressed proteins.
